# Supplementary material for: Lithography for robust and editable atomic-scale silicon devices and memories
Source: Nat Commun. 2018 Jul 23;9:2778. doi: 10.1038/s41467-018-05171-y (PMC6056515; doi:10.1038/s41467-018-05171-y)
Supplement: Supplementary file 1 — Supplementary Information [file 41467_2018_5171_MOESM1_ESM.pdf]

Supplementary Information

Lithography for Robust and Editable Atomic-scale Silicon Devices and Memories

Roshan Achal[1,2*], Mohammad Rashidi[1,2], Jeremiah Croshaw[1], David Churchill[3], Marco Taucer[1,2], Taleana Huff[1,2], Martin Cloutier[4], Jason Pitters[2,4], Robert A. Wolkow[1,2,4]

[*]Correspondence to: achal@ualberta.ca

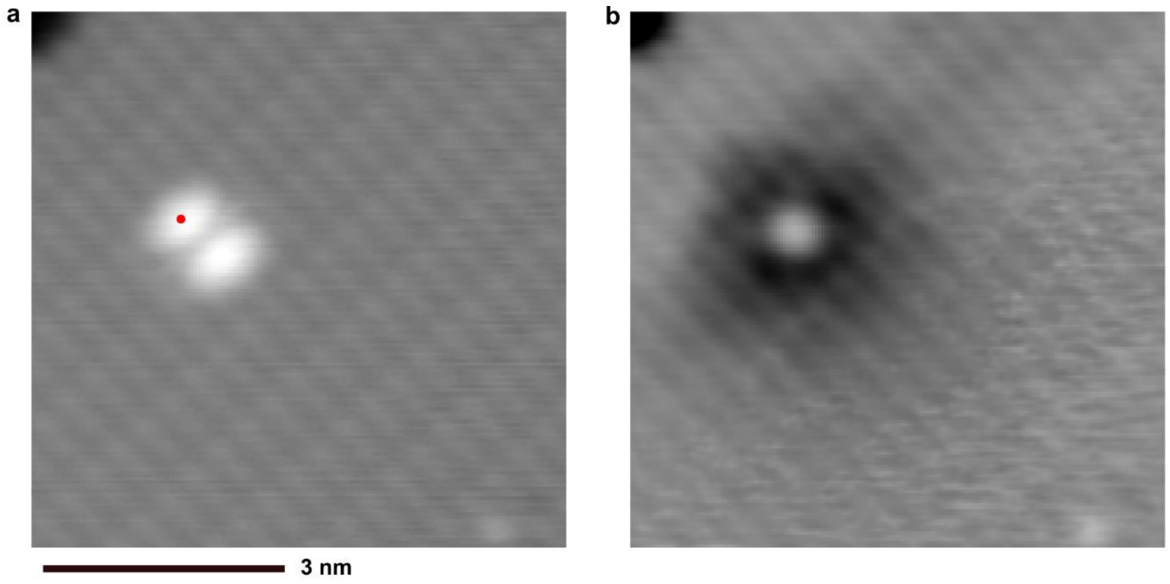

Supplementary Figure 1. Atomically precise hydrogen repassivation of one dangling bond in a pair.

a, A Scanning Tunneling Microscope (STM) image (V=1.4 V, I=50 pA, T=4.5 K, 6.5 $\times$ 6.5 nm^2) of two dangling bonds (DBs) on the hydrogen-passivated Si(100)-2x1 surface at the closest possible spacing, commonly known as a bare dimer structure. An STM tip, with adsorbed hydrogen on its surface, is positioned above the site denoted with a red dot for repassivation. **b**, The STM image immediately following the hydrogen repassivation of the site in **a**. Only the desired DB has been repassivated, leaving the other unaltered, illustrating the precise nature of this technique.

Supplementary Note 1

Atomic hydrogen

On rare occasions during hydrogen lithography we observe atomic hydrogen physisorbing onto the sample surface after the creation of a dangling bond (DB), instead of the assumed action of traveling into vacuum[1,2]. It has been found that instead of sticking to the sample surface there is a probability it may absorb to the tip surface instead[1]. The atomic hydrogen can be dragged by the Scanning Tunneling Microscope (STM) tip, where in Supplementary Figure 2c it has been moved away from the DB towards the middle of the structure. The STM tip can be reliably functionalized with this atom for the purpose of repassivation[1], or to remove it from the area where a structure is being built.

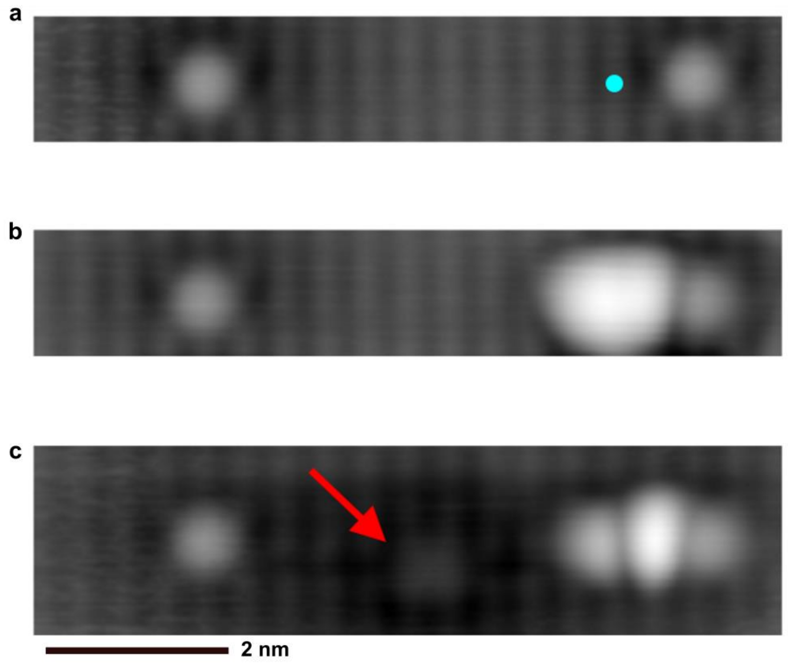

Supplementary Figure 2. Atomic hydrogen physisorption on hydrogen-passivated silicon.

a, (V=1.4 V, I=50 pA, T=4.5 K, 1.7 × 8 nm^2) A two dangling bond (DB) structure with the location of the next site for hydrogen lithography denoted with a blue circle. **b**, The DB structure after an attempted hydrogen removal, it appears an error has been made as the site in **a**, does not resemble a single DB, instead appearing brighter, as if multiple hydrogens have been removed from the surface. **c,** After repeated scans over the structure the error was no longer present and a faint object (red arrow) was now visible. The object is atomic hydrogen that physisorbed to the sample surface after extraction instead of traveling into vacuum[1]. The hydrogen atom was altering the appearance of the newly created DB in **b**, causing it to look like an error had occurred. The hydrogen atom is able to sit in close proximity to the DB in **b**, without rebonding. A description of the feature in between the two DBs on the right-hand side in **c**, has been proposed elsewhere[3].

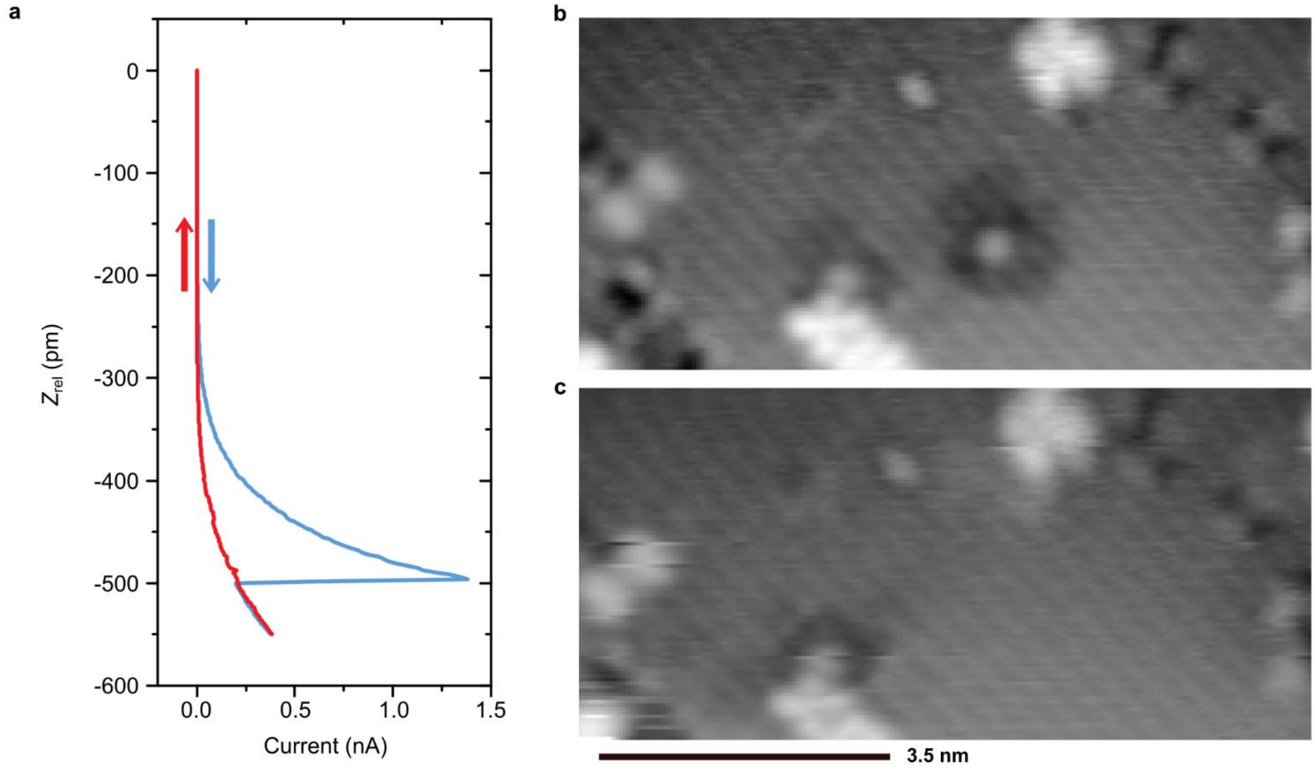

Supplementary Figure 3. Hydrogen repassivation with type-II signature at 77 K.

a, (V=0.2 V) Type-II signature in the Scanning Tunneling Microscope (STM) tunneling current recorded during hydrogen repassivation (HR) at 77 K. **b-c**, (V=1.4 V, I=50 pA, T=77 K, 4.5×9.3 nm^2) STM images before and after successful HR.

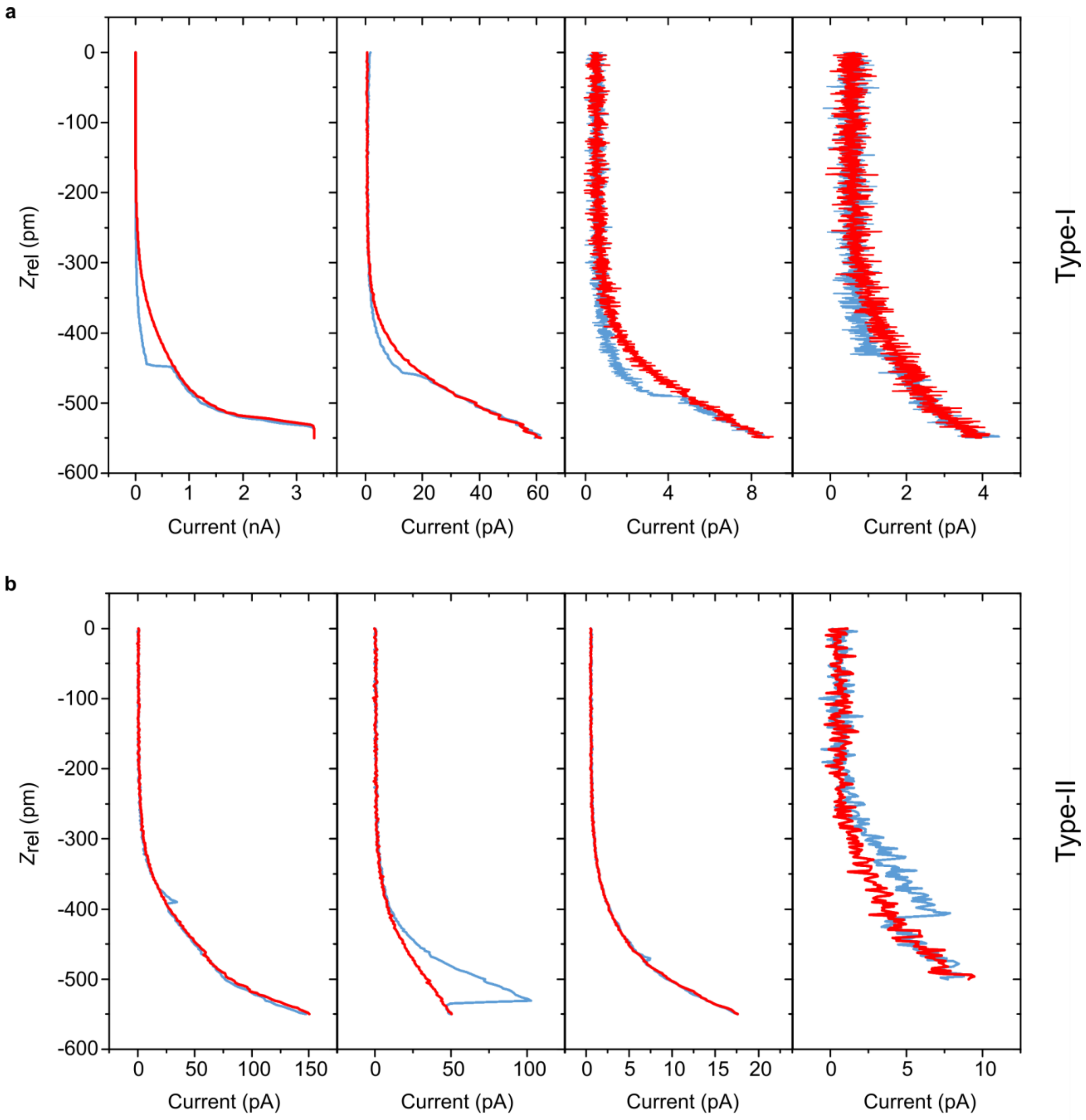

Supplementary Figure 4. Taxonomy of type-I and type-II hydrogen repassivation signatures.

a-b, A representative sample of different type-I and type-II signatures recorded during hydrogen repassivation (HR) events (T=4.5 K), with the tip set over a dangling bond at 1.4 V and 50 pA. Applied bias voltages in **a**, from left to right, are 0.5 V, 0.3 V, 0.2 V, 0.2 V respectively. Applied bias voltages in **b**, from left to right, are 0.2 V, 0.4 V, 0.2 V, 0.2 V respectively. While the magnitude of both signatures can vary depending on the apex orbital and the choice of applied voltage to limit the tunneling current during HR, their overall shapes remain very characteristic. The reliability and reproducibility of these features makes them excellent indicators of successful repassivation for automation routines.

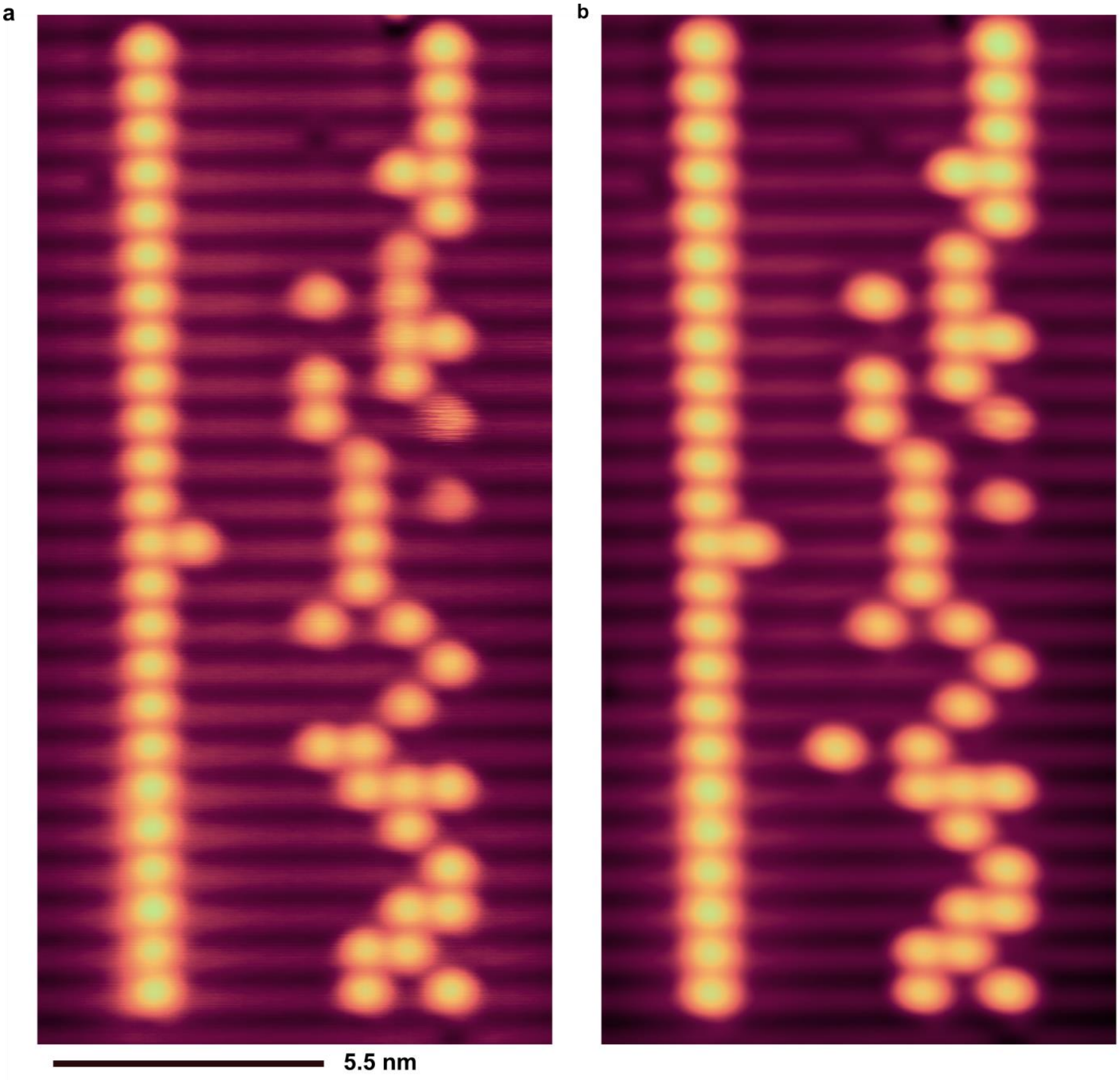

Supplementary Figure 5. Stability and Rewriting of the 192-bit memory.

a, (V=-1.75 V, I=50 pA, T=4.5 K, 21.5 × 10.7 nm^2)The original memory structure created through hydrogen lithography (HL) and hydrogen repassivation (HR). The pattern was initially entered into the HL program incorrectly through human error. As a result, one of the lines was incorrectly written. **b**, The same memory 72 hours later with the incorrect line rewritten using HR, no additional changes were made to the structure. The cryo-fluid in the Scanning Tunneling Microscope had to be replenished during this time, so the memory was subjected to temperature spikes up to 12 K and pressure spikes on the order of 10^{-8} Torr.

Supplementary Movie 1

Playback of musical information stored in the 192-bit memory from a Scanning Tunneling Microscope image.

Supplementary References

1. Huff, T. R. *et al.* Atomic White-Out: Enabling Atomic Circuitry Through Mechanically Induced Bonding of Single Hydrogen Atoms to a Silicon Surface. *ACS Nano* **11,** 8636–8642 (2017).

2. Ballard, J. B. *et al.* Spurious dangling bond formation during atomically precise hydrogen depassivation lithography on Si(100): The role of liberated hydrogen. *J. Vac. Sci. Technol. B, Nanotechnol. Microelectron. Mater. Process. Meas. Phenom.* **32,** 021805 (2014).

3. Schofield, S. R. *et al.* Quantum engineering at the silicon surface using dangling bonds. *Nat. Commun.* **4,** 1649 (2013).
