# Supplementary material for: Lithography for robust and editable atomic-scale silicon devices and memories
Source: Nat Commun. 2018 Jul 23;9:2778. doi: 10.1038/s41467-018-05171-y (PMC6056515; doi:10.1038/s41467-018-05171-y)
Supplement: Supplementary file 2 — Description of Additional Supplementary Files [file 41467_2018_5171_MOESM2_ESM.pdf]

Description of Additional Supplementary Files

File Name: Supplementary Movie 1

Description: Readout of a 192-bit atomic-scale memory from a Scanning Tunneling Microscope image. The information stored in the 192-bit memory can be read directly as the Scanning Tunneling Microscope (STM) scans over the structure or from previously recorded STM images, as in this case (V=-1.8 V, I=50 pA, T=4.5 K, 21.5 × 10.7 nm^2). Image recognition software is used to identify the presence of dangling bonds (DBs) in fixed locations on the surface to determine if the value of a bit is zero (no DB present) or one (DB present). Each line of the memory consists of 8 bits.
